# Treating medically unexplained symptoms via improving access to psychological therapy (IAPT): major limitations identified

**DOI:** 10.1186/s40359-020-0380-2

**Published:** 2020-02-05

**Authors:** Keith Geraghty, Michael J. Scott

**Affiliations:** 1 Centre for Primary Care, Division of Health Sciences and Population Health, University of Manchester, Manchester, UK; 2 Liverpool, UK

**Keywords:** Improving access to psychological therapies, Medically unexplained symptoms, Chronic fatigue syndrome, Myalgic encephalomyelitis, Recovery measurement, Treatment

## Abstract

**Background:**

Improving Access to Psychological Therapies is a UK Government funded initiative to widen access to psychological treatment for a range of common mental health complaints, such as depression and anxiety. More recently, the service has begun to treat patients with medically unexplained symptoms. This paper reports on a review of treatment protocols and early treatment data for medically unexplained symptoms, specifically the illness myalgic encephalomyelitis/chronic fatigue syndrome.

**Main text:**

A series of seven core problems and failings are identified, including an unproven treatment rationale, a weak and contested evidence-base, biases in treatment promotion, exaggeration of recovery claims, under-reporting of drop-out rates, and a significant risk of misdiagnosis and inappropriate treatment.

**Conclusions:**

There is a pressing need for independent oversight of this service, specifically evaluation of service performance and methods used to collect and report treatment outcomes. This service offers uniform psycho-behavioural therapy that may not meet the needs of many patients with medically unexplained health complaints. Psychotherapy should not become a default when patients’ physical symptoms remain unexplained, and patients should be fully informed of the rationale behind psychotherapy, before agreeing to take part. Patients who reject psychotherapy or do not meet selection criteria should be offered appropriate medical and psychological support.

## Background: IAPT brief

One of the most significant recent developments in UK mental health service delivery has been the introduction and rollout of an NHS initiative called ‘Improving Access to Psychological Therapy’ (IAPT). This programme started in 2007–8 with the Secretary of State for Health allocating £173 million from the comprehensive spending review to fund the first 3 years of IAPT. IAPT is an initiative to expand mental health service provision or at least address the unmet needs of mental health care in the UK, as identified in Lord Richard Layard’s Prime Minister’s Strategy Unit seminar held at the London School of Economics in 2004, entitled ‘Mental Health: Britain’s Biggest Social Problem’ [1]. IAPT is said to be a stepped -provider of soft mental care services for patients with mild to moderate mental health problems [1], such as anxiety and depression. Patients with more severe or acute mental health needs are to be treated as usual within NHS mental health services. IAPT also treats panic disorder, obsessive compulsive disorder, social phobia, post-traumatic stress and more recently, medically unexplained symptoms (MUS) [2].

The inclusion of MUS patients under the IAPT remit signifies a massive expansion of IAPT coverage, far beyond patients with common mental health complaints, to patients with complex medical complaints [2, 3]. IAPT literature lists chronic fatigue syndrome (also known as myalgic encephalomyelitis) and irritable bowel syndrome as the main medically unexplained symptom disorders it will treat ([2], Section 2 p10–11), but other conditions may fall within this category, such as chronic back pain and unexplained headaches. Many millions of patients seen in primary care fall under the MUS label. IAPT has already begun to treat patients with MUS and long-term medical conditions via 37 early implementer sites. Between 2008 and 2011, IAPT trained and employed 3600 psychological therapists, with plans to expand numbers [2]. Psychotherapists are mainly trained in offering cognitive behavioural therapy (CBT), a psychotherapy intervention developed by Aaron Beck in the 1960s to treat depression [4]. There are plans to locate IAPT therapists in GP practices and acute medical centres, including accident and emergency departments, to offer onsite access to psychological therapies [2, 3].

The rationale for IAPT offering CBT as the treatment for MUS is threefold. First, its mandate is to provide what it calls ‘evidence-based psychological therapies’ [5] and CBT is a recommended treatment for myaglic encephalomyelitis/chronic fatigue syndrome by the UK National Institute of Health Care Excellence (NICE) [6], thus IAPT seeks to provide CBT services to ME/CFS patients and other MUS categories. Second, IAPT retains the services of academic advisors, many of whom promote CBT as a treatment of MUS. Primary care doctors (GPs) are encouraged to refer MUS patients to IAPT, and patients can self-refer [1, 7]. Third, promoters of IAPT argue that there will be a cost saving (economic rationale), that IAPT treatments reduce spending on front-line community medical support, expensive secondary specialist care and disability/social benefits [3, 5].

## Main text

### Difficulties defining MUS

Medically unexplained symptoms refer to persistent bodily complaints for which adequate examination (including investigation) does not reveal sufficiently explanatory structural or other specified pathology [2, 8]. MUS are also referred to as ‘functional somatic syndromes’; bodily complaints such as dizziness, fatigue, pain, headaches and so on, that remain unexplained [9]. Myaglic encephalomyelitis or chronic fatigue syndrome (ME/CFS) is one of the most cited medically unexplained symptom illnesses [9]. Myalgic encephalomyelitis (ME) is a post-infectious disease causing malaise, muscle weakness, and nervous system complaints, primarily pain, cognitive dysfunction, and sleep disturbance [10], whereas chronic fatigue syndrome (CFS) is an alternative label introduced in the late 1980s to describe a pattern of symptoms, specifically unexplained fatigue [11]. Other MUS illness categories are symptom-based, such as chronic headaches or unexplained back pain. Henningsen writes,

“*There is no objective criterion to decide whether a pattern of bodily complaints should be seen as a functional somatic syndrome or as indicator of a medically explained disease or as something else, and lists drawn up by different authors reflect their particular backgrounds and views*” [9] p546.

Essentially, doctors decide to ascribe a diagnosis of medically unexplained symptom once other illnesses are excluded, and experts from different fields, psychiatry, primary care, neurology and so on, apply different rationale to classify MUS. Doctors tend to put MUS-type patients under code-categories such as idiopathic pain, tiredness or gastrointestinal complaints, rather than under an MUS code-category, making research in this area complex. In 2013, the American Psychiatric Association rejected the term ‘medically unexplained symptoms’ in its *Diagnostic and Statistical Manual* (DSM-5) and replaced it with the related construct of somatization with “somatic symptom disorder” [12]; which refers ‘to excessive thoughts, feelings, or behaviours related to the somatic symptoms or health concerns’. One problem that immediately comes to the fore is that most disorders deemed MUS by IAPT, such as ME/CFS or IBS, are often not considered somatoform or somatisation disorders by experts and DSM-5 states ‘It is not appropriate to give an individual a mental disorder diagnosis solely because a medical cause cannot be demonstrated’ [12].

### A problematic treatment model

IAPT literature often refers to a cognitive behavioural (CB) model of medically unexplained symptoms [2, 5]. This ‘CB model of MUS’ is set out in a paper by Deary et al., that discusses research around aetiology of MUS and the use of CBT as treatment modality [13]. This model focuses on hypothesised events in MUS pathogenesis, set out under three headings: 1. Predisposing factors, 2. Precipitating factors and 3. Perpetuating factors. These 3-Ps are also intertwined into a grand biopsychosocial model [14] of illness. Despite the inclusion of ‘biological factors’, the CB model is heavily focused on exploration of psychological factors claimed to ‘perpetuate’ MUS, such as personality factors like perfectionism, illness beliefs (attributions), cognitions (catastrophising), and behaviours such as symptom focusing (somatising) and avoiding activity (due to fear-avoidance or anxiety) [15–17]. It is noteworthy that IAPT employs academic advisors who promote the CB Model of MUS [13, 15]. This may partly explain why IAPT recommends CBT as a treatment for MUS and long-term medical conditions (LTCs) [18]. However, in illnesses like multiple sclerosis or diabetes (LTCs), the illness itself is not said to be perpetuated by psychological factors, but symptoms, such as fatigue or pain, are tentatively linked to anxiety, personality factors and avoidant behaviours [19, 20].

In MUS, the role of CBT is to directly alter ‘unhelpful’ cognitions or behaviours said to perpetuate symptoms in a ‘vicious cycle’ [13]. The cognitive behavioural model of MUS of Deary et al., draws from a wide variety of literature and evidence concerning the efficacy of CBT in randomised controlled trials, with much emphasis on ME/CFS treatment evidence. It is not feasible for us to consider all this evidence within the confines of this paper however, this evidence is critiqued elsewhere [21, 22]**.**

Deary et al. is cited as the seminal paper for MUS treatment by Kellett et al. in their review of MUS IAPT service performance (section 5) [23]. However, Deary et al. do not offer a robust analysis of the evidence for MUS treatment. Deary et al. write, “*There are varying degrees of evidence for each of the components of this model. What is lacking is solid proof of their interaction in vicious circles, although all the models reviewed assume this interaction*” [13]. Essentially, their model of MUS is speculative, and the cycle used to frame their model remains theoretical. Deary et al. also state,

“*What makes the CBT model so difficult to test may also be one of its chief strengths: it is in many ways a meta-model, providing a skeleton structure to join the dots of whatever factors each patient presents…This means that every client will have, in effect, their own model, making the testing of a generic CBT MUS model impossible*” [13] p788.

The CB model of MUS is identical to the CB model of chronic fatigue syndrome [24] - we note that the MUS treatment model is derived from theory and research in ME/CFS. Yet, Deary et al. fail to mention the widespread opposition of ME/CFS patients and advocacy groups to this treatment model and the psychological framing of the illness [25, 26]. This we view as a bias, promotion of CBT as a treatment for MUS without discussion of negative patient feedback or critical literature.

### A mixed evidence-base

Clark states that IAPT treatments are ‘evidence-based’ [3]. However, the Deary et al. [13] review of evidence for MUS treatment using CBT finds null to moderate size effects for reducing somatic symptoms: benefits are often small to moderate using CBT. A recent systematic review of cognitive behavioural interventions in MUS also shows weak benefits in reducing healthcare use [27]. This is not a strong evidence-base for IAPT to treat MUS. The question then arises, should IATP be seeking to treat patients with medically unexplained symptoms based on a small number of CBT clinical trials that show mixed results with modest benefits?

A review of the literature on the treatment of patients with MUS in primary care by Edwards et al. reveals that different studies use different labels and criteria to investigate MUS: ‘somatisation and symptom syndrome’, ‘somatoform disorders’, ‘irritable bowel syndrome’, ‘common somatic symptoms’, and ‘medically unexplained symptoms’ [28]. It is doubtful that such labels identify one homogenous group of patients, making extrapolation of treatment benefits highly problematic. Other terms have been proposed for MUS, including persistent physical symptoms (PPS) or functional somatic syndrome (FSS). MUS is a moving target, a label given to a patient with unexplained medical complaints. Another difficulty in applying the literature to the full range of MUS patients is that most trials compare usual care (usually no care or continuing GP care) with one-to-one talk therapies like CBT. The design of these trials tends to favour CBT and there are strong therapy effects, placebo and expectancy effects, to consider [29]. Edwards et al., also point out that a wide range of treatments appear to help patients with MUS, not just CBT – essentially most interventions out-perform usual care, even self-help guides [28].

In practice, community doctors encounter a considerable percentage of patients with unexplained symptoms – a 45% rate is mentioned in the MUS literature to emphasise the ‘MUS problem or dilemma’ [8]. However, this is not the same as the actual rate for persistent MUS, given a patient is rarely diagnosed on first visit to a GP. In clinical practice, a patient often takes months to years to be diagnosed with MUS; NICE recommends a diagnosis of ME/CFS after a minimum of 4 months of unexplained fatigue and many patients take years to receive a diagnosis [30]. Estimates of rates of persistent physical symptoms complaints in primary care are much lower (3–10%) [31, 32]. Aamland et al. [31] find that the commonest MUS complaints are musculoskeletal problems and unexplained fatigue. If we then consider that IAPT wishes to treat these patients, we begin to see a problem: that IAPT therapists will now be asked to manage patients with chronic back pain, headaches, unexplained fatigue, or unexplained pain, using psychotherapy only. All forms of medical care, such as physiotherapy for back pain or analgesics for chronic pain, give way to CBT.

The exact causes of IBS and ME/CFS are not established, but this is not the same as saying these illnesses remain ‘medically unknown’; there is a vast body of literature on both disorders. This then begs the question, what does ‘medically unexplained’ really mean? Should a physician tell a patient they have IBS or MUS? In most cancers for instance, exact causation remains relatively uncertain and in autoimmune illnesses like lupus or rheumatoid arthritis, causation and pathogenesis often remain obscure. There is no clear line to demarcate the MUS patient IAPT appears to want to treat, from the medical patient. IAPT therapists are being asked to treat some of the most challenging and chronically ill patients in modern clinical medicine. ME/CFS patients for example, have quality of life scores far below patients with multiple sclerosis, cancer, and other major life illnesses [33]. Yet, ME/CFS patients do not receive anything like the type of medical care MS or cancer patients receive. MS and cancer patients may be offered CBT if they experience depression or anxiety, but not instead of medical care, but in addition to it; whereas in MUS or ME/CFS, psychotherapy appears to exist as the main NHS treatment pathway on offer.

### ME/CFS: the headline MUS disorder

The science on ME/CFS is rapidly evolving with a vast amount of evidence pointing to a post-infectious type illness [34, 35]. Most ME/CFS patients recount that their illness started after an infection and many studies are beginning to show neuro-immunological and cellular abnormalities in ME/CFS [36–38]. However, front-line doctors do not have accessible tests to discern ME/CFS, thus it is an illness diagnosed by exclusion of other conditions – which may explain why IAPT consider ME/CFS an MUS. Treatment of ME/CFS remains problematic given a lack of consensus on aetiology and pathogenesis. Over the past few decades, cognitive behavioural therapy and graded exercise therapy (GET) have been tested on ME/CFS patients with modest benefits reported [39].However, this evidence has been robustly criticised [40] with post-hoc analysis of trial evidence showing the added benefits from CBT to be negligible [22, 41, 42].

There appears to be two divergent models of the illness evident in the literature: contemporary versions of Ramsay’s myalgic encephalomyelitis, a post-infectious disease of nerve, brain and muscle [43], versus a model derived from psychiatry of chronic fatigue syndrome as a biopsychosocial manifestation of neurasthenia or affective disorders [44]. These divergent explanatory models include divergent treatment approaches. Ramsay advocated rest, pacing and medical interventions, whereas Wessely and others advocate CBT and GET interventions and the minimisation of medical investigations [13, 45, 46]. IAPT accepts the Wessely paradigm over Ramsay’s disease. The Deary et al. [13] model of MUS emerges from the psychiatric model of ME/CFS [24] and underpins the IAPT treatment approach [47]. IAPT references a small number of RCTs of CBT-GET that have shown modest benefits for ME/CFS patients with mild to moderate symptoms [39, 48–50]. However, such trials have no blinding (a cornerstone of the RCT), often focus on subjective outcomes (how much better a participant states they feel at the end of the trial), lack objective measures and use few controls. Other trials of CBT-GET have shown little substantive benefits in ME/CFS [51, 52]. In addition, the modest added benefits reported in some RCTs, fall away over the long-term [53]. RCTs of CBT-GET do not include patients with severe illness presentations, those housebound or bedbound. These inherent biases and weaknesses in trial evidence are not mentioned in IAPT literature.

If we look at NHS clinical data, in one study of adult ME/CFS patients treated with CBT in specialist units, Collin et al. found that while approximately 1/3rd of ME/CFS patients report some benefits from CBT, only 5.7% of patients counted themselves as no longer having ME/CFS after treatment (based on 435 patients followed up for 1 year post-CBT) [54]. Essentially, most patients reported no benefits and 90% + continue to report ME/CFS after CBT. In another study of GP referrals of patients with ME/CFS to a specialist centre in Newcastle, Newton et al. found that up to 40% of patients referred had other medical and mental health conditions (not ME/CFS) [55]. Geraghty et al. conducted an analysis of ME/CFS patient survey data spanning 15 years and found that the majority of patients do not report CBT to be helpful, most find GET unhelpful or detrimental, and in contrast the biggest percentage report self-pacing or guided pacing to be most beneficial [56]. The above evidence does not appear in IAPT literature on MUS treatment or ME/CFS treatment.

### IAPT early performance data and recovery measurement

A Kellett et al. IAPT service review for early providers of treatments for MUS reports significant levels of success [23], however this review reveals CBT treatment for MUS is based on depression and anxiety symptoms only. The Kellett et al. evaluation uses the Patient Health Questionnaire-9 (PHQ-9) to measure depression [57] (PHQ-9 severity ratings, range 0–27, with a cut-off score for the detection of depression being a score ≥ 10) and the GAD-7 to measure generalized anxiety disorder [58] (GAD-7 severity ratings, range 0–21, a cut-off score ≥ 8 detects an anxiety disorder with adequate sensitivity and specificity). Depression and anxiety may be secondary or co-morbid complaints in MUS conditions like ME/CFS, IBS, or fibromyalgia, thus any improvement in scores, whilst welcomed, do little to validate CBT as a primary treatment for MUS, where the primary problems in ME/CFS for example, are physical and social impairment as a result of fatigue, pain or orthostatic intolerance. Unger et al. found that ME/CFS patients’ SF-36 (short-form) scores differed least for mental health complaints and most on physical function, bodily pain and social function [59]. Why does IAPT not use these indicators as the main markers to benchmark recovery?

**Fig. 1:**
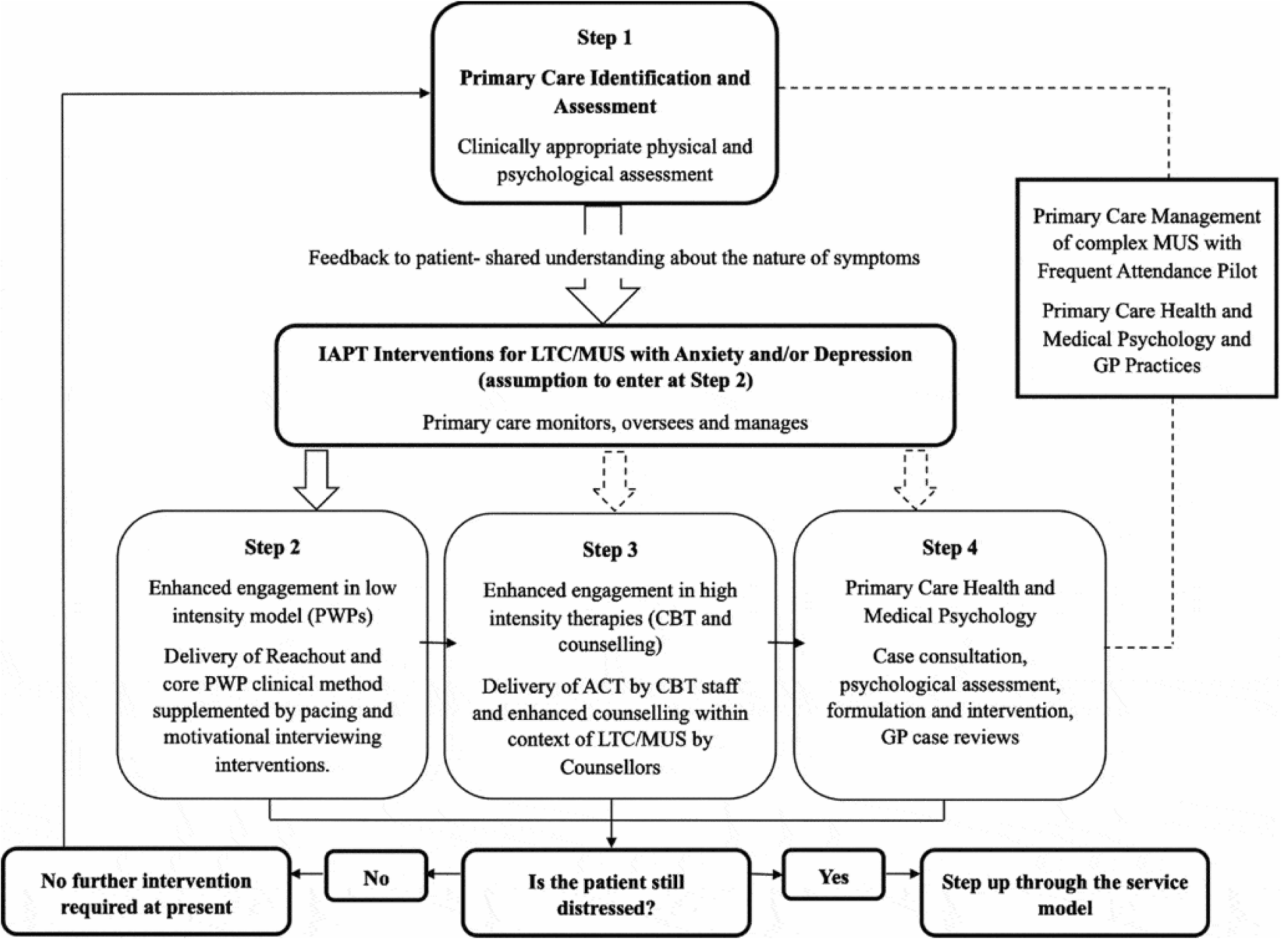
Stepped care psychological service model for LTMCs/MUS IAPT Pathfinder Pilot

**Fig. 2:**
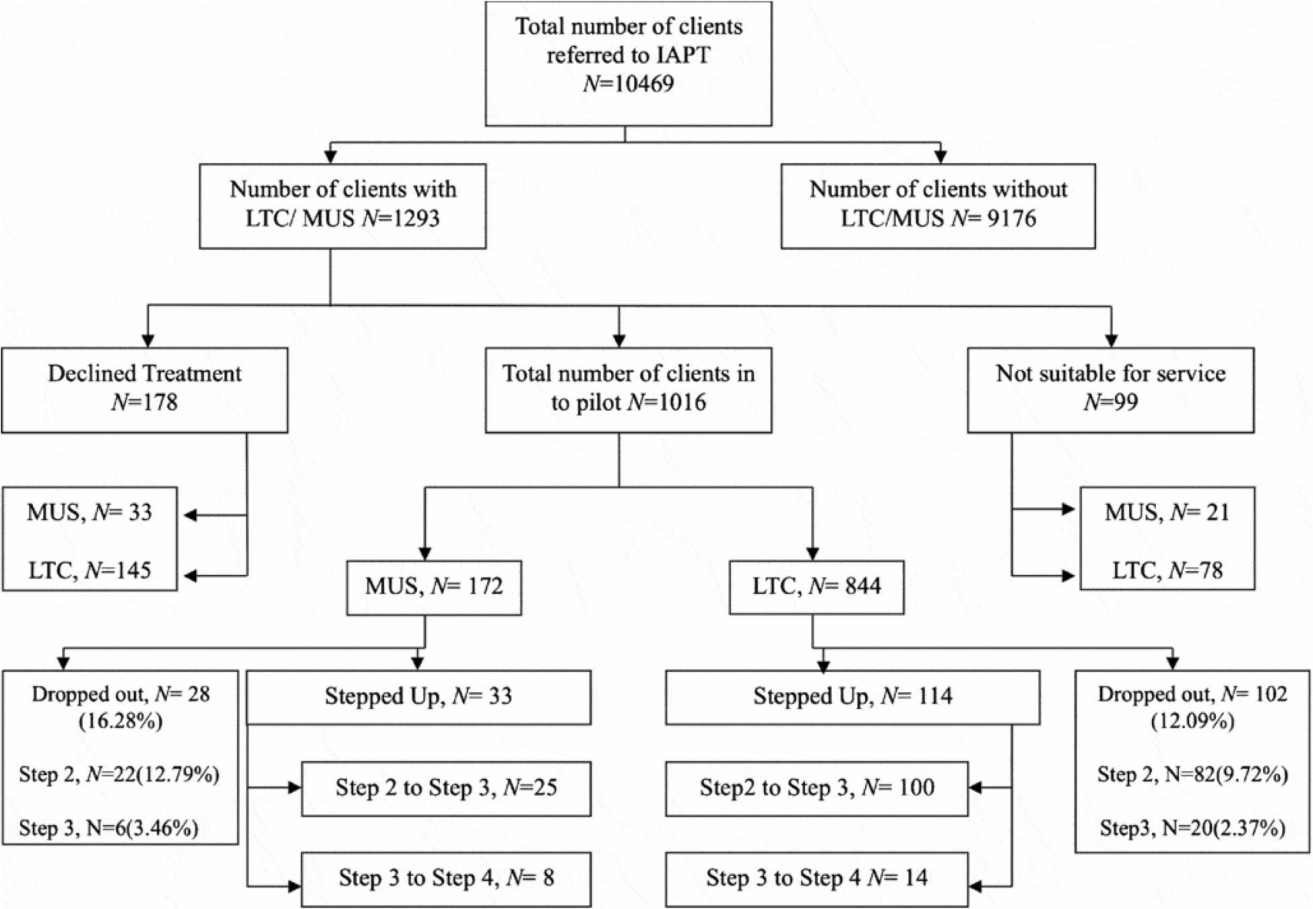
Patient flow through the IAPT LTC/MUS stepped-care model

We reviewed Kellet et al. data and found that just 172 MUS patients were treated out of 10,469 referred to IAPT with possible long-term conditions (LTCs) or MUS (Figs. 1 and 2). At step 1 of treatment for MUS, only 33 patients are given further care, and by step 3 & 4, only 8 receive full treatment with CBT. Data is given that 28 dropped out at step 1, 22 dropped out at step 2 and 6 dropped out after step 3 [23]. Seventy-eight patients with MUS dropped out during treatment stages and 61 dropped out before treatment or did not complete treatment. This means 45.25% of MUS patients treated by IAPT do not complete treatment or drop out after entering treatment (not counting the patients IAPT reject as inappropriate MUS patients). Only 4.6% of the MUS patients IAPT takes into treatment, complete intensive CBT.

Kellet et al., report two types of improvement; ‘limited’ and ‘reliable’ and what they term ‘moving to recovery’. (A) *moving to recovery*: patients above the clinical cut-off before intervention and below following intervention. A patient was a “case” when they scored above the clinical threshold on depression and/or anxiety before intervention (i.e. PHQ-9 score ≥ 10 at assessment and/or GAD-7 ≥ 8 at assessment); whereas moving to recovery is when the final outcome score was below the clinical threshold on depression and anxiety (i.e. PHQ-9 score < 10 at termination and GAD-7 < 8 at termination) [23]. Moving to recovery rates for depression were 32.33% for LTC and 29.35% for MUS. Moving to recovery rates for anxiety were 30.43% for LTC and 29.03% for MUS. (B) *reliable improvement* requires that any improvement in outcome scores pre and post intervention exceeded measurement error of the PHQ-9 and GAD-7 using reliable change criteria. Reliable improvement was a reduction of ≥6 points on the PHQ-9 or ≥ 4 points on the GAD-7 [23]. Reliable improvement rates for depression were LTCs (39.07%) and MUS (33.70%) and for anxiety LTCs (47.14%) and MUS (45.16%). The overall reliable recovery rate for MUS is 17.39%, while deterioration rates were 4.32% for MUS [23]. We do not know whether patients with MUS improve their primary medical problems and somatic symptoms, pain, fatigue and so on. We know a high number of patients drop out of treatment, circa 45%, leaving an overly positive cohort for improvement analysis. Also, IAPT does not record the specific types of conditions the MUS patients presented with.

### Therapist competency and risk of misdiagnosis

The IAPT workforce of therapists are trained in high intensity CBT and low intensity interventions, such as telephone advice for patients [5]. The training of IAPT therapists follows a CBT curriculum formulated by IAPT advisors (Table 1 in Appendix). For example, in ME/CFS treatment the CBT offered by IAPT is aligned with the type of CBT tested in clinical trials of the CB model [39, 48, 60]. The training curriculum is linked to core competencies therapists need to acquire [61]. IAPT CBT trainees undertake a year-long course with 1 day per week of formal training and additional in-practice supervision.

Looking at Table 1 in Appendix competencies, we identify problem areas. IAPT therapists may well be able to understand the aetiology and epidemiology of MUS disorders like ME/CFS, however to what extent are IAPT therapists equipped to make a differential diagnosis in ME/CFS, fibromyalgia or irritable bowel syndrome? Grouping patients with unexplained symptoms into a broad category (MUS) carries a high risk of misdiagnosis. This concern is observed in a review of 418 CFS patient referrals to a specialist chronic fatigue clinic, where 37% of referrals were rejected as inappropriate, and of these, 61% had a likely alternative diagnosis [62]. In a follow-up survey of patients assessed in-clinic, 43% had an alternative medical or psychiatric diagnosis [62]. This is similar to the 40% diagnostic error rate reported by Newton et al., where many patients were eventually diagnosed with other conditions, 47% with a chronic disease, 20% a primary sleep disorder, 15% a psychological/psychiatric illness (most commonly, depression, anxiety and post-traumatic stress disorder), and 4% a cardiovascular disorder [55]. IAPT therapists will not be equipped to discern true or false cases of MUS. Patients referred to IAPT may have legal grounds to bring claims against the NHS if misdiagnosed. There is a concern that IAPT therapists focused on depression and anxiety complaints may miss deterioration in patients with underlying physical complaints, or wrongly interpret worsening of physical symptoms, as signs of anxiety or stress.

### The patient-therapist relationship and informed consent

Doctors, particularly GPs, report feeling stressed dealing with MUS patients, particularly ME/CFS [63]. There is a degree of hubris in the notion that some IAPT therapists, with no specialist medical or advanced psychological training, will be equipped to manage the complex medical problems many MUS patients present with. The CB model IAPT therapists are being trained to employ may benefit some patients with MUS, particularly any suffering depression and anxiety. However, many MUS patients may not benefit and are likely to experience anger and frustration at being referred for psychotherapy [64]. IAPT therapists will have to manage this conflict.

The cognitive behavioural model of MUS is arguably more prescriptive than the CBT model of depression devised by Beck [4]. In Beck’s theory, CBT is used to help the patient explore beliefs about themselves and their thoughts, such as self-loathing. The therapist helps the patient explore reasons for this and strategies to develop a sense of self-worth. In the Deary et al. [13] model of MUS, the patient is to be challenged on the origins of symptoms such as pain or fatigue - the patient is said to perpetuate their own illness by holding on to beliefs in an ‘organic’ illness (ME/CFS caused by infection for example). The notion patients’ beliefs perpetuate illnesses such as ME/CFS, IBS or Fibromyalgia, is based more on speculation than evidence. In contrast, there is considerable growing evidence showing ME/CFS is indeed linked to biological dysfunction following infection [34, 35]. The IAPT model of MUS may put CBT therapist and patient on a collision course – far from Beck’s collaborative journey. To what extent will the CB Model of MUS be disclosed to patients remains to be seen [41]. We speculate many MUS patients will be told very little about the therapies they will receive in IAPT, before agreeing to participate. We further speculate that if the rationale behind CBT is disclosed to MUS patients, many will reject treatment or withdraw from treatment – we see high dropout rates in early IAPT provider data [23].

## Discussion

The provision of access to mental health services is an initiative most academics and clinicians support. However, given the £1 billion+ investment commitment to the UK IAPT service, it is appropriate to evaluate the remit and performance of IAPT. Mental health has historically received less investment than other areas of medicine and IAPT is seen as a shift in a positive direction. Few critical articles exist in the literature on the work of IAPT. IAPT recently publicised a 50% recovery rate in treating headline mental health complaints [2, 3]. However, Scott has shown that the true recovery rate may be closer to 10% [65]. In response, Binnie writes that while it is laudable to critique IAPT recovery statistics, it may be unwise to criticise IAPT, given IAPT is the only service outlet for distressed patients not well served by traditional medical services [66]. This may be true for patients with a range of mental health complaints, but it is not a transferable argument for patients with medically unexplained symptoms. ME/CFS patients for instance, reject psychiatric framing of the illness [25] and many perceive cognitive behavioural therapies to be both unhelpful and harmful [41, 56, 67]. Such facts should not be ignored by IAPT supporters.

An important finding from this paper concerns the selection of MUS patients and the application of psycho-behavioural therapies. GPs are being encouraged to refer MUS patients deemed to have depression and anxiety: “*All referrals were made by GPs who recognized the need for psychological intervention due to mental health issues (i.e. anxiety and depression) being implicated in poor LTC/MUS self-management*” ([23] p555). IAPT targets MUS patients with mild to moderate depression and anxiety issues, while MUS patients with complex medical needs, or without affective disorders, are not accepted by IAPT. IAPT uses changes in depression and anxiety scores as indicators of improvement and recovery in MUS. We see in Kellet et al. that from 10,469 patients referred to IAPT for treatment, only 172 patients were eligible for MUS treatment and of these 25 patients were moved to step 2 (received self-help guidance, motivational interviewing or pacing manual), while only 8 patients received step 3–4 care (intensive CBT) – a tiny number.

IAPT does not fully record dropouts, non-completed treatment, or patients rejecting treatment – resulting in positive treatment bias. IAPT only records cases that complete specific steps in treatment – grossly distorting improvement and recovery rate data. IAPT is also failing to measure improvement in symptoms that define MUS conditions, such as fatigue, sleep disturbance, physical and social function in ME/CFS, or gastric complaints in IBS, or bodily pain in fibromyalgia.

IAPT employs a MUS treatment model [13] that has been devised by IAPT advisors. This model originates from the cognitive behavioural model of ME/CFS [24]; it is mostly theoretical and lacks strong evidential support. MUS symptoms are claimed to be perpetuated by patients holding unhelpful beliefs about their symptoms or adopting avoidance behaviours. The aim of therapy is to challenge these beliefs/behaviours. However, IAPT treatment also encompasses self-management, pacing, and counselling-type support [23]. In ME/CFS, surveys reveal that patients find ‘pacing’ the most appropriate approach to illness coping, above CBT or graded exercise therapy [56]. IAPT supporters often recount the success of CBT, without mention of the use of non-CBT based interventions, such as counselling or lifestyle advice.

If CBT helps some MUS patients minimise psychological distress, anxiety and depression (often co-morbid complaints to chronic health conditions), this does not mean patients no longer suffer MUS. Interestingly, even the staunchest promoters of a CB model acknowledge that these treatments are non-curative: Wessely writes, “*Clinical researchers and funding agencies would note that, even though these interventions appear effective, the evidence is based on a small number of studies and neither approach is remotely curative, and would continue their efforts to develop better treatments*” ([68], p1378). It is arguable that CBT is an adjunct support therapy that perhaps helps a percentage of patients with MUS express their fears, distress and frustration, in a clinical setting. If so, this may partly explain modest benefits found in clinical trials. However, we observe that recovery is operationally defined by IAPT in a way that will not resemble how most MUS patients understand recovery – for instance, recovery from chronic back pain should mean much reduced or non-existent back pain, rather than less depression or anxiety [69].

We know from clinical data that very few ME/CFS patients recover using CBT [54, 56]. Collin et al. found that just 5.7% of patients seen in NHS specialist CBT clinics, with more expertise than IAPT centres, report no longer having ME/CFS after treatment [54]. It is a rather misguided view to assume that IAPT therapists will be able to treat all MUS patients. Additionally, IAPT therapists do not need to be clinical psychologists, thus are unlikely to be able to assess ongoing or emergent mental health complaints. The IAPT treatment model is likely to lead to considerable distress for many patients with MUS complaints that endure post-IAPT, or who reject such treatments as inappropriate for them [64]. Will these patients be returned to GP care, when GPs were unable to help them overcome their MUS complaint in the first instance? What then, for the patient with MUS?

Some leading UK clinicians characterise MUS as a major cost problem to the NHS and a challenge to GPs [8], with reference to studies that suggest MUS account for up to 45% of GP consultations [70]. If correct (we think not), IAPT would collapse under the weight of MUS patients – *a reductio ad absurdum*. The fact GPs need to refer large swathes of patients to IAPT indicates a failing in contemporary medicine – how to accommodate the needs of patients with medically unexplained complaints. Despite the strong dictates to refer MUS patients to IAPT, clearly this does not mean all MUS patients but those hand-picked that meet IAPT screening protocols for depression or anxiety issues; whilst unwanted MUS patients are to be returned to primary care doctors, with no alternative medical care pathway in sight due to the MUS label – with both patient and doctor left frustrated.

## Conclusions

IAPT has a mandate to improve access to psychological therapies in the UK NHS. Most health professionals support this service, particularly for common mental health complaints, where there is considerable unmet need. However, IAPT is now seeking to treat chronic medical conditions and medically unexplained illnesses. First, given the vast number of patients with possible MUS, this will not be feasible. Second, conditions that fall under the MUS label, such as ME/CFS or IBS, are not exclusively medically unexplained. There is considerable emerging evidence elucidating the pathophysiology of ME/CFS as a possible neuro-immune disease. The IAPT treatment rationale for MUS rests on a weak and contested cognitive behavioural model that is promoted by IAPT advisors. Many patients with MUS conditions, particularly ME/CFS, are unlikely to benefit from attending IAPT. This leads us to ask whether IAPT should be seeking to expend considerable scarce resources on treating MUS. IAPT therapists are not equipped to manage patients with complex medical conditions. High rates of misdiagnosis observed to date are unlikely to be corrected by IAPT therapists. High drop-out rates are to be expected and many patients may return to GPs feeling distressed by inappropriate referral for CBT. IAPT needs to improve its reporting mechanisms and overhaul its formula for determining improvement and recovery in MUS. Current methods inflate recovery statistics. IAPT services could benefit from independent oversight and auditing.

## Data Availability

Not applicable.

## Appendix

**Table 1:** Competences to be Demonstrated by IAPT Therapists after training on ME/CFS

UNIT 3.3: Chronic Fatigue Syndrome (CFS) / Myalgic Encephalopathy (ME) Aims: To be able to demonstrate knowledge of evidence-based interventions for people with Chronic Fatigue Syndrome, and practical skills in their application.	
• Ability to draw on knowledge of the aetiology, epidemiology and presentation of CFS/ME, and of its differential diagnosis (and exclusion criteria) • Ability to draw on knowledge of factors considered to contribute to the development of • CFS/ME (including physical illness/ serious infections (such as glandular fever), lifestyle, stress, perfectionism and distress)17 • Ability to draw on knowledge of factors considered to maintain CFS/ME (such as patterns of activity characterised by boom and bust cycles, unhelpful fear avoidance beliefs leading to avoidance of activity), attentional biases towards symptoms) and how these link to physiological mechanisms including poor sleep and deconditioning • Ability to help client feel that their experience of CFS/ME is being listened to and respected (i.e. acknowledging that they are experiencing real, physical symptoms) • Ability to conduct a comprehensive assessment of the client’s symptoms, including their medical and prescription history, contextual information and main current difficulties, physical symptoms, patterns of activity and rest, coping mechanisms, the impact of CFS/ME on their life and specific concerns about symptoms, fears about engaging in activity, attentional focus and how significant others respond to symptoms • Ability to introduce the CBT model, collaboratively identifying predisposing and precipitating factors and a vicious cycle of fatigue • Ability to introduce and discuss planning activity and rest in the context of short and long term activity targets (establishing a consistent approach to activity initially before gradually increasing activity levels) • Ability to ensure that a focus on graded exercise is integrated into the intervention • Ability to help the client monitor sleep, identify specific sleep problems that exacerbate fatigue and discuss sleep strategies such as an up time and bed restriction • Ability to employ attentional training to address symptom focussing • Ability to work on unhelpful thoughts related to engaging in activity more consistently and perfectionism, generate alternatives and help the client test these out with gradual behaviour change and behavioural experiments • Ability to identify and work with potential obstacles to recovery • Ability to use standard and idiosyncratic measures to evaluate outcomes with CBT for CFS • Ability to help clients prepare for ending therapy and maintain improvements by identifying possible indicators of relapse and strategies for their management	

Source: Improving Access to Psychological Therapies: National Curriculum for CBT in the Context of Long Term Persistent and Distressing Health Conditions Version 20, June 2017. (accessed Oct 2018) https://www.hee.nhs.uk/sites/default/files/documents/CBT%20LTC%20MUS%20curriculum.pdf

## Abbreviations

CB: Cognitive behavioural
CBT: Cognitive behavioural therapy
GET: Graded exercise therapy
IAPT: Improving access to psychological therapies
ME: Myalgic encephalomyelitis
MUS: Medically unexplained symptoms
NHS: National Health Service
NICE: Institute of Health Care Excellence

## References

[1] Holland R (2009). Improving access to psychological therapies: the intention. London J Prim Care.

[2] NHS (2018). The improving access to psychological therapies manual.

[3] Clark DM (2018). Realizing the mass public benefit of evidence-based psychological therapies: the IAPT program. Annu Rev Clin Psychol.

[4] Beck AT. Depression: clinical, experimental, and theoretical aspects: University of Pennsylvania Press; 1967.

[5] Clark DM (2011). Implementing NICE guidelines for the psychological treatment of depression and anxiety disorders: the IAPT experience. Int Rev Psychiatry.

[6] Baker R, Shaw EJ (2007). Diagnosis and management of chronic fatigue syndrome or myalgic encephalomyelitis (or encephalopathy): summary of NICE guidance. BMJ.

[7] Gibson JC, Smith B, Ward CD (2011). Chronic fatigue syndrome. InnovAiT.

[8] Chew-Graham CA, Heyland S, Kingstone T, Shepherd T, Buszewicz M, Burroughs H (2017). Medically unexplained symptoms: continuing challenges for primary care. Br J Gen Pract.

[9] Henningsen P, Zipfel S, Herzog W (2007). Management of functional somatic syndromes. Lancet.

[10] Ramsay AM, Dowsett EG, Dadswell JV, Lyle WH, Parish JG (1977). Icelandic disease (benign myalgic encephalomyelitis or Royal Free disease). BMJ.

[11] Holmes GP (1988). Chronic fatigue syndrome: a working case definition. Ann Intern Med.

[12] Association AP. Diagnostic and statistical manual of mental disorders (DSM-5®): American Psychiatric Pub; 2013. 10.1590/s2317-17822013000200017 24413388

[13] Deary V, Chalder T, Sharpe M (2007). The cognitive behavioural model of medically unexplained symptoms: a theoretical and empirical review. Clin Psychol Rev.

[14] Engel G (1977). The need for a new medical model: a challenge for biomedicine. Science.

[15] Deary V, Chalder T (2010). Personality and perfectionism in chronic fatigue syndrome: a closer look. Psychol Health.

[16] Knoop H, Prins JB, Moss-Morris R, Bleijenberg G (2010). The central role of cognitive processes in the perpetuation of chronic fatigue syndrome. J Psychosom Res.

[17] Hulme K, Hudson JL, Rojczyk P, Little P, Moss-Morris R (2017). Biopsychosocial risk factors of persistent fatigue after acute infection: a systematic review to inform interventions. J Psychosom Res.

[18] Dennison L, Moss-Morris R (2010). Cognitive-behavioral therapy: what benefits can it offer people with multiple sclerosis?. Expert Rev Neurother.

[19] Moss-Morris R, Dennison L, Yardley L, Landau S, Roche S, McCrone P (2009). Protocol for the saMS trial (supportive adjustment for multiple sclerosis): a randomized controlled trial comparing cognitive behavioral therapy to supportive listening for adjustment to multiple sclerosis. BMC Neurol.

[20] Jopson NM, Moss-Morris R (2003). The role of illness severity and illness representations in adjusting to multiple sclerosis. J Psychosom Res.

[21] Geraghty K, Jason L, Sunnquist M, Tuller D, Blease C, Adeniji C (2019). The ‘cognitive behavioural model’ of chronic fatigue syndrome: critique of a flawed model. Health Psychol Open.

[22] Wilshire CE, Kindlon T, Courtney R, Matthees A, Tuller D, Geraghty K (2018). Rethinking the treatment of chronic fatigue syndrome-a reanalysis and evaluation of findings from a recent major trial of graded exercise and CBT. BMC Psychol.

[23] Kellett S, Webb K, Wilkinson N, Bliss P, Ayers T, Hardy G (2016). Developing services for patients with depression or anxiety in the context of long-term physical health conditions and medically unexplained symptoms: evaluation of an IAPT pathfinder site. Behav Cogn Psychother.

[24] Surawy C, Hackmann A, Hawton K, Sharpe M (1995). Chronic fatigue syndrome: a cognitive approach. Behav Res Ther.

[25] Spandler H, Allen M. Contesting the psychiatric framing of ME/CFS. Soc Theory Health. 2017.

[26] Blease C, Carel H, Geraghty K. Epistemic injustice in healthcare encounters: evidence from chronic fatigue syndrome. J Med Ethics. 2016. 10.1136/medethics-2016-103691 27920164

[27] Jones B (2019). de C Williams AC. CBT to reduce healthcare use for medically unexplained symptoms: systematic review and meta-analysis. Br J Gen Pract.

[28] Edwards TM, Stern A, Clarke DD, Ivbijaro G, Kasney LM (2010). The treatment of patients with medically unexplained symptoms in primary care: a review of the literature. Ment Health Fam Med.

[29] Blease CR (2015). Talking more about talking cures: cognitive behavioural therapy and informed consent. J Med Ethics.

[30] Cairns R, Hotopf M (2005). A systematic review describing the prognosis of chronic fatigue syndrome. Occup Med (Oxford, England).

[31] Aamland A, Malterud K, Werner EL (2014). Patients with persistent medically unexplained physical symptoms: a descriptive study from Norwegian general practice. BMC Fam Pract.

[32] Verhaak PF, Meijer SA, Visser AP, Wolters G (2006). Persistent presentation of medically unexplained symptoms in general practice. Fam Pract.

[33] Falk Hvidberg M, Brinth LS, Olesen AV, Petersen KD, Ehlers L (2015). The health-related quality of life for patients with myalgic encephalomyelitis / chronic fatigue syndrome (ME/CFS). PLoS One.

[34] Green CR, Cowan P, Elk R, O'Neil KM, Rasmussen AL (2015). National Institutes of Health pathways to prevention workshop: advancing the research on myalgic encephalomyelitis/chronic fatigue syndrome. Ann Intern Med.

[35] Komaroff AL (2015). Myalgic encephalomyelitis/chronic fatigue syndrome: a real illness. Ann Intern Med.

[36] Montoya JG, Holmes TH, Anderson JN, Maecker HT, Rosenberg-Hasson Y, Valencia IJ (2017). Cytokine signature associated with disease severity in chronic fatigue syndrome patients. Proc Natl Acad Sci U S A.

[37] Hornig M, Montoya JG, Klimas NG, Levine S, Felsenstein D, Bateman L (2015). Distinct plasma immune signatures in ME/CFS are present early in the course of illness. Sci Adv.

[38] Komaroff AL, Takahashi R, Yamamura T, Sawamura M (2018). Neurologic abnormalities in myalgic encephalomyelitis/chronic fatigue syndrome: a review. Brain Nerve.

[39] White PD, Goldsmith KA, Johnson AL, Potts L, Walwyn R, DeCesare JC (2011). Comparison of adaptive pacing therapy, cognitive behaviour therapy, graded exercise therapy, and specialist medical care for chronic fatigue syndrome (PACE): a randomised trial. Lancet.

[40] Twisk FN, Maes M (2009). A review on cognitive behavorial therapy (CBT) and graded exercise therapy (GET) in myalgic encephalomyelitis (ME) / chronic fatigue syndrome (CFS): CBT/GET is not only ineffective and not evidence-based, but also potentially harmful for many patients with ME/CFS. Neuro Endocrinol Lett.

[41] Geraghty KJ, Blease C (2018). Cognitive behavioural therapy in the treatment of chronic fatigue syndrome: a narrative review on efficacy and informed consent. J Health Psychol.

[42] Geraghty KJ (2017). Further commentary on the PACE trial: biased methods and unreliable outcomes. J Health Psychol.

[43] Ramsay AM (1957). Encephalomyelitis in North West London. Lancet.

[44] Wessely S (1994). Neurasthenia and chronic fatigue: theory and practice in Britain and America. Transcult Psychiatr Res Rev.

[45] Wessely S, Sharpe M, Hotopf M. Chronic fatigue and its syndromes: Oxford University Press; 1998.

[46] Halligan P, Aylward M. The power of belief: psychosocial influences on illness, disability and medicine: Oxford University Press; 2006.

[47] Moss-Morris Rona, Deary Vincent, Castell Bronwyn (2013). Chronic fatigue syndrome. Neurological Rehabilitation.

[48] Deale A, Chalder T, Marks I, Wessely S (1997). Cognitive behavior therapy for chronic fatigue syndrome: a randomized controlled trial. Am J Psychiatry.

[49] Sharpe M, Hawton K, Simkin S, Surawy C, Hackmann A, Klimes I (1996). Cognitive behaviour therapy for the chronic fatigue syndrome: a randomized controlled trial. BMJ.

[50] Moss-Morris R, Sharon C, Tobin R, Baldi JC (2005). A randomized controlled graded exercise trial for chronic fatigue syndrome: outcomes and mechanisms of change. J Health Psychol.

[51] Lloyd AR, Hickie I, Brockman A, Hickie C, Wilson A, Dwyer J (1993). Immunologic and psychologic therapy for patients with chronic fatigue syndrome: a double-blind, placebo-controlled trial. Am J Med.

[52] Wearden AJ, Dowrick C, Chew-Graham C, Bentall RP, Morriss RK, Peters S (2010). Nurse led, home based self help treatment for patients in primary care with chronic fatigue syndrome: randomised controlled trial. BMJ.

[53] Sharpe M, Goldsmith KA, Johnson AL, Chalder T, Walker J, White PD (2015). Rehabilitative treatments for chronic fatigue syndrome: long-term follow-up from the PACE trial. Lancet Psychiatry.

[54] Collin SM, Crawley E (2017). Specialist treatment of chronic fatigue syndrome/ME: a cohort study among adult patients in England. BMC Health Serv Res.

[55] Newton JL, Mabillard H, Scott A, Hoad A, Spickett G (2010). The Newcastle NHS chronic fatigue syndrome service: not all fatigue is the same. J R Coll Physicians Edinb.

[56] Geraghty KJ, Hann M, Kurtev S. ME/CFS patients’ reports of symptom changes following CBT, GET and pacing treatments: analysis of a primary survey compared with secondary surveys. J Health Psychol. 2017. 10.1177/1359105317726152 28847166

[57] Kroenke K, Spitzer RL, Williams JBW (2001). The PHQ-9. J Gen Intern Med.

[58] Spitzer RL, Kroenke K, Williams JW, Löwe B (2006). A brief measure for assessing generalized anxiety disorder: the gad-7. Arch Intern Med.

[59] Unger ER, Lin JS, Tian H, Natelson BH, Lange G, Vu D (2017). Multi-site clinical assessment of myalgic encephalomyelitis/chronic fatigue syndrome (MCAM): design and implementation of a prospective/retrospective rolling cohort study. Am J Epidemiol.

[60] Sharpe M (1998). Cognitive behavior therapy for chronic fatigue syndrome: efficacy and implications. Am J Med.

[61] Roth AD, Pilling S (2008). Using an evidence-based methodology to identify the competences required to deliver effective cognitive and Behavioural therapy for depression and anxiety disorders. Behav Cogn Psychother.

[62] Devasahayam A, Lawn T, Murphy M, White PD (2012). Alternative diagnoses to chronic fatigue syndrome in referrals to a specialist service: service evaluation survey. JRSM Short Rep.

[63] Raine R (2004). General practitioners’ perceptions of chronic fatigue syndrome and beliefs about its management, compared with irritable bowel syndrome: qualitative study. BMJ.

[64] Geraghty KJ, Blease C. Myalgic encephalomyelitis/chronic fatigue syndrome and the biopsychosocial model: a review of patient harm and distress in the medical encounter. Disabil Rehabil. 2018:1–10. 10.1080/09638288.2018.1481149 29929450

[65] Scott MJ (2018). Transforming improving access to psychological therapies. J Health Psychol.

[66] Binnie J (2018). Medical approaches to suffering are limited, so why critique improving access to psychological therapies from the same ideology. J Health Psychol.

[67] Geraghty KJ, Esmail A (2016). Chronic fatigue syndrome: is the biopsychosocial model responsible for patient dissatisfaction and harm?. Br J Gen Pract.

[68] Wessely S (2001). Chronic fatigue syndrome—trials and tribulations. JAMA.

[69] Devendorf AR, Jackson CT, Sunnquist M, Jason LA. Defining and measuring recovery from myalgic encephalomyelitis and chronic fatigue syndrome: the physician perspective. Disabil Rehabil. 2017:1–8. 10.1080/09638288.2017.1383518 PMC6123286 28982247

[70] Haller H, Cramer H, Lauche R, Dobos G. Somatoform disorders and medically unexplained symptoms in primary care: a systematic review and meta-analysis of prevalence. Dtsch Arztebl Int. 2015. 10.3238/arztebl.2015.0279 PMC4442550 25939319

